# PTH induced osteoblast Slit3 to decrease aberrant sensory innervation in degenerated vertebral endplates to relieve low back pain in mice

**DOI:** 10.1038/s41413-025-00488-z

**Published:** 2026-01-22

**Authors:** Weixin Zhang, Arryn D. Otte, Zhuolun Wang, Sisir Kumar Barik, Mei Wan, Xu Cao, Janet L. Crane

**Affiliations:** 1 Department of Orthopedic Surgery, Johns Hopkins University School of Medicine, Baltimore, MD USA; 2 Division of Pediatric Endocrinology, Johns Hopkins University School of Medicine, Baltimore, MD USA; 3 Department of Neurology, Johns Hopkins University School of Medicine, Baltimore, MD USA

**Keywords:** Bone, Endocrine system and metabolic diseases

## Abstract

During aging, the spine undergoes degenerative changes, particularly with vertebral endplate bone expansion and sclerosis, that are associated with nonspecific low back pain. We report that parathyroid hormone (PTH) treatment reduced vertebral endplate sclerosis and improved pain behaviors in three mouse models of spinal degeneration (aged, SM/J, and young lumbar spine instability mice). Aberrant innervation in the vertebral body and endplate during spinal degeneration was decreased with PTH treatment as quantified by PGP9.5^+^ and CGRP^+^ nerve fibers, as well as CGRP expression in dorsal root ganglia. The neuronal repulsion factor Slit3 significantly increased in response to PTH treatment mediated by transcriptional factor FoxA2. PTH type 1 receptor and Slit3 deletion in osteocalcin-expressing cells prevented PTH-reduction of endplate porosity and improvement in behavior tests. Altogether, PTH stimulated osteoblast production of Slit3, decreased aberrant sensory nerve innervation, and provided symptomatic relief of LBP associated with mouse spinal degeneration.

## Introduction

Low back pain (LBP) is one of the most common medical complaints. Chronic LBP profoundly affects quality of life and daily physical activity and is a crucial risk factor for future health decline.^1–3^ Most LBP is nonspecific with no apparent pathoanatomical cause aside from intervertebral disc (IVD) degeneration.^4–7^ Studies indicate that the prevalence of LBP peaks between the ages of 40 and 69 years, affecting 28%–42% of individuals. In the USA, the annual cost associated with LBP management surpasses 100 billion dollars.^8^ The primary therapeutic approaches include behavioral management, pharmacological treatments like non-steroidal anti-inflammatory drugs or muscle relaxants, and surgical interventions, all aimed at maintaining function,^9^ but fail to address the underlying pathophysiology. A notable pathological feature of LBP is aberrant nociceptive innervation of the spine, impacting structures such as muscles, ligaments, IVD, and vertebral endplate.^10^ The vertebral endplate, positioned between the IVD and vertebral body, is normally composed of a thin layer of hyaline cartilage and participates in shock absorption for each spinal unit.^11,12^ In recent years, we have demonstrated that during aging or with mechanical instability, vertebral endplates undergo degeneration, expanding in size as the endplate becomes calcified, porous, and aberrantly innervated. Specifically, as osteoclasts remodel the calcified endplate generating pores, they create an acidic environment, become senescent and secrete factors, such as Netrin-1, that induce sensory innervation, causing LBP.^13–18^ Elimination of senescent cells with a senolytic drug significantly decreases sensory innervation and subsequently reduces LBP.^16,17^

Aging of the musculoskeletal system results in chronic skeletal pain, especially in conditions such as osteoarthritis and spinal degeneration.^19–21^ Pain is a process by which noxious stimuli are converted into electrical signals by different receptors or channels in specialized sensory neurons called nociceptors.^22,23^ Once the nociceptor is sufficiently activated, the electrical signal is transmitted along the nerve fibers towards the spinal cord and brain.^24^ As the pain signal travels, its strength and character can be modulated by various factors in different regions.^25,26^ In recent years, the regulation of nociceptive innervation in mediating hypersensitivity in osteoarthritis and spinal degeneration has emerged as the concept of skeletal interoception, a process where internal organs sense and transmit information to the central nervous system.^27^ Beyond the sensitization of sensory nerve fibers by inflammatory stimuli and osteoclast secretion of Netrin-1 during skeletal degeneration, nociceptors in the subchondral bone and sclerotic endplate have increased expression of the receptor of Netrin-1, Deleted in Colorectal Cancer (DCC), amplifying pain signaling.^13,28^ Synthesis of prostaglandin E2 (PGE2) is triggered in the degenerated joint and upregulates the sodium channel Nav 1.8 in the nociceptors.^11^ Therapies that block the PGE2 pathway, whether through cyclooxygenase-2 (COX-2) inhibitors or sensory nerve blockade, notably reduce pain.^14^

Parathyroid hormone (PTH), produced and secreted by the parathyroid glands, plays an essential role in the regulation of calcium and phosphate metabolism, as well as bone metabolism.^29^ Intermittent administration of PTH primarily stimulates bone formation, whereas continuous elevation of PTH significantly promotes bone resorption.^30^ Our research has shown that PTH treatment impacts not only bone structural remodeling but also alleviates osteoarthritic pain and spinal hypersensitivity in animal models by promoting osteoblastic bone formation in the porous endplates and reducing aberrant innervation and PGE2 concentrations.^31–34^ However, the mechanism by which PTH treatment reduces sensory innervation in the porous endplates remains unclear. During development, the distribution of nerve fibers is orchestrated by various guiding factors. These factors ensure that nerve fibers, also known as axons, navigate accurately to their designated targets, thereby establishing functional neural circuits. The primary guiding factors include Netrins, Slits, Semaphorins, Ephrins, Neurotrophins, and others, which can be secreted by diverse sources, such as neurons, endothelial cells, immune cells, osteoblasts, and osteoclasts.^15,35,36^ The mechanisms by which guiding factors regulate sensory innervation or denervation and their subsequent influence on pain in skeletal diseases, such as LBP, remain elusive. In the current research, we found that PTH stimulated the production of Slit3 in osteoblast lineage cells, which was associated with a reduction in aberrant sensory innervation and improvement in spinal hypersensitivity behaviors associated with LBP.

## Results

### Degenerated endplate structure and pain behavior are improved by PTH treatment

**Fig. 1:**
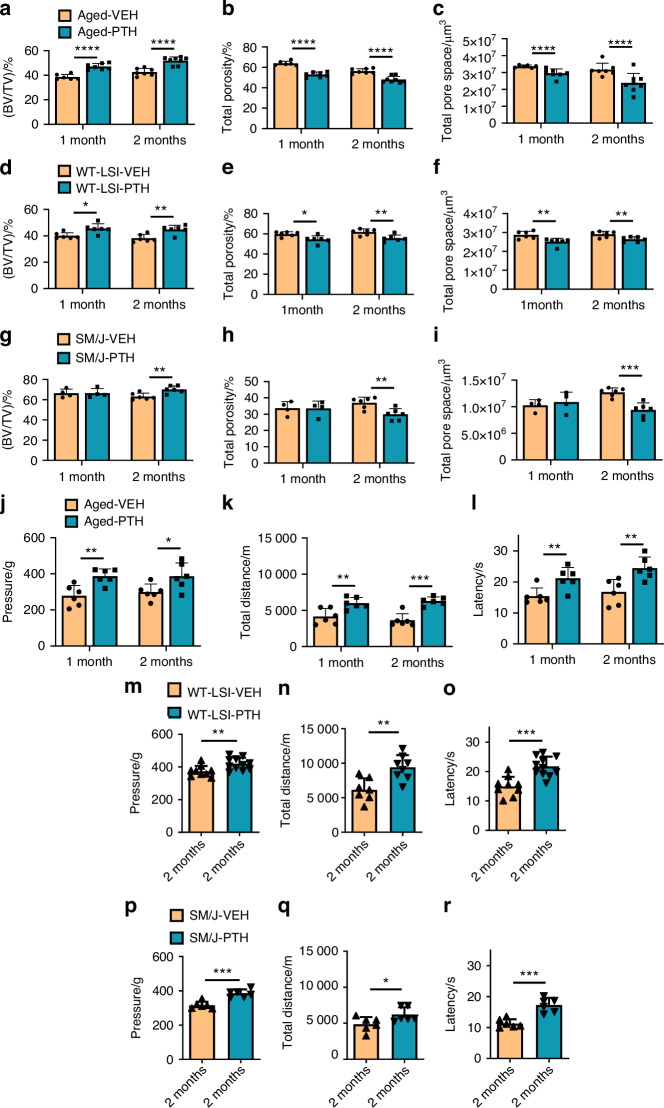
Micro-CT and behavior analysis of three spinal degeneration mouse models. Vertebral endplate bone structure analyses by micro-computed tomography: Bone volume/Tissue volume (BV/TV) (**a**), Total porosity percentage (**b**) and Total pore space (**c**) of fifth lumbar (L5) endplate of aged mice treated with parathyroid hormone (PTH) (40 µg/kg/d) for 1 or 2 months relative to vehicle-treated (Veh) group. (*n* ≥ 6, *t*-test). BV/TV (**d**), Total porosity percentage (**e**) and Total pore space (**f**) of L5 endplate of WT young mice 2 months after lumbar spine instability (LSI) surgery and treated with PTH (40 µg/kg/d) for 1 or 2 months relative to Veh-group. (*n* ≥ 6, *t*-test). BV/TV (**g**), Total porosity percentage (**h**) and Total pore space (**i**) of L5 endplate of SM/J mice treated with PTH (40 µg/kg/d) for 1 or 2 months relative to Veh-group. (*n* ≥ 4, *t*-test). Behavior evaluations included pressure tolerance in the lumbar spine region as determined by force threshold (**j**), total distance covered during spontaneous activity in 2 days (**k**), and latency of hind paw withdrawal post-thermal stimulation (**l**) in aged mice treated with PTH or Veh for 1 or 2 months. (*n* ≥ 6, *t*-test). Pressure tolerance in the lumbar spine region (**m**), total distance covered during spontaneous activity in 2 days (**n**), and latency of hind paw withdrawal post-thermal stimulation (**o**) in WT young mice 2 months after LSI surgery and treated with PTH or Veh for 2 months. (*n* ≥ 8, *t*-test). Pressure tolerance in the lumbar spine region (**p**), total distance covered during spontaneous activity in 2 days (**q**), and latency of hind paw withdrawal post-thermal stimulation (**r**) in SM/J mice treated with PTH or Veh for 2 months. (*n* ≥ 6, *t*-test). *P < 0.05, **P < 0.01, ***P < 0.005, ****P < 0.001

To assess the efficacy of PTH treatment regarding LBP, we utilized 3 spinal degeneration models: (1) aged C57BL/6J (WT) mice (22 months of age) to mimic natural aging,^37^ (2) young wild type (WT) mice (2–3 months of age) two months after lumbar spine instability (LSI) surgery to mimic mechanical instability^12^ (Fig. S1a), and (3) SM/J transgenic mice (6 months of age) to mimic accelerated aging due to underlying genetic causes.^38^ Over the duration of 2 weeks, 1 or 2 months, mice were administered PTH (40 µg/kg/d) or vehicle daily via intraperitoneal injection. Therefore, the final endpoint age of assessment for animal models was 24 months (aged), 6 months (LSI), and 8 months (SM/J) (Fig. S1a). Bone microarchitecture of the fifth lumbar spine endplate (L5) was evaluated using micro-computed tomography (micro-CT). Our findings revealed significant changes in the spine endplate morphology by 1 month for both the aged and LSI mouse models, whereas similar changes were not observed until after 2 months of PTH treatment in the SM/J model relative to vehicle controls. Specifically, there was a significant increase in bone volume and decreased total porosity and pore space of the L5 endplate in the PTH group relative to the vehicle group (Fig. 1a–i and Fig. S1b–g). To ascertain whether PTH treatment alleviated chronic spinal pain in our experimental models, we subjected aged, WT LSI, and SM/J mice to a series of behavior tests, including hyperalgesia to applied pressure, spontaneous wheel running, and thermal tolerance. In aged mice, PTH treatment for durations of 1 and 2 months, but not 2 weeks, showed a notable improvement in hyperalgesia pressure tolerance of lumbar spine and in the total distance of spontaneous running on the active-wheel (Fig. 1j, k and Fig. 1h). All three PTH treatment durations significantly extended the latency of the hind paw withdraw to the thermal stimulation by Hargreaves test in aged mice relative to vehicle (Fig. 1l and Fig. S1i). Behavior tests in WT LSI and SM/J mice after 2 months of PTH administration demonstrated significant improvement in pressure tolerance, total distance on spontaneous wheel running, and enhanced the tolerance to thermal stimulation relative to vehicle controls (Fig. 1m–r). Collectively, these observations underscore that PTH treatment can mitigate chronic spinal pain and improve the degenerated endplate structure across various mouse models of spinal degeneration.

### Nociceptive innervation decreased in PTH-treated mice

**Fig. 2:**
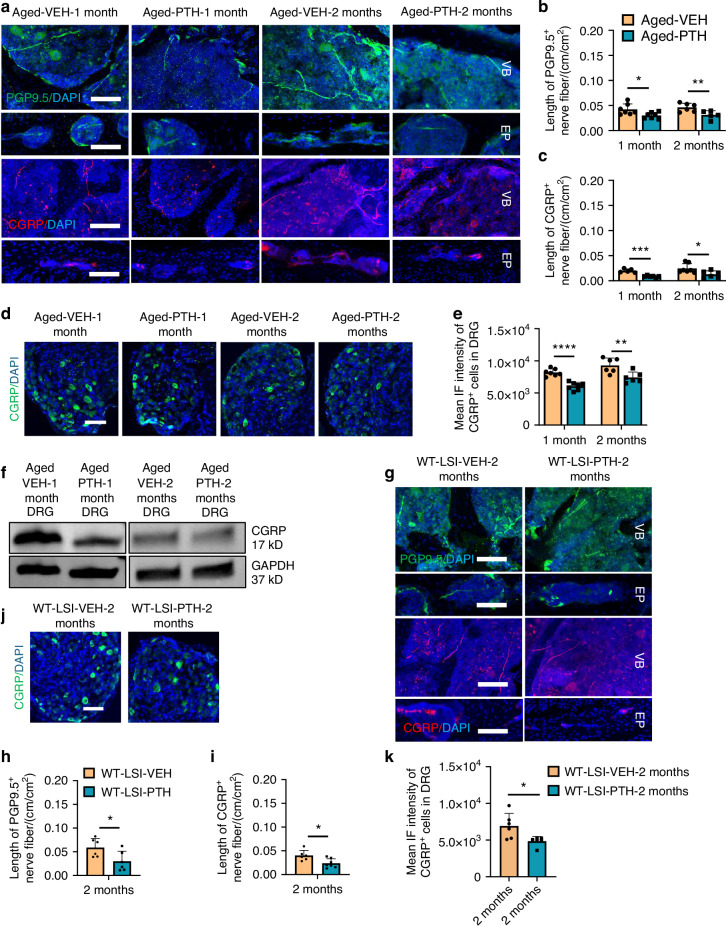
PTH treatment alters innervation in the degenerated spine. **a** Representative images of PGP9.5-positive fibers (upper row, Green) and CGRP-positive fibers (lower row, Red) in the lumbar vertebral body and endplate of aged mice treated with PTH or Veh for 1 or 2 months. Scale bar: 100 µm. Quantitative analysis of the length of PGP9.5-positive fibers (**b**) or CGRP-positive fibers (**c**) in the lumbar vertebral body of aged mice treated with PTH or Veh for 1 month or 2 months (*n* ≥ 5, *t*-test). Representative images (**d**) and quantitative mean immunofluorescent (IF) intensity (**e**) of CGRP-positive neurons in the dorsal root ganglia (DRG) (L1-L2) of aged mice treated with PTH or Veh for 1 month or 2 months. Scale bar: 100 µm. (*n* ≥ 6, *t*-test). **f** Protein levels of CGRP in DRG tissue (L1–L2) relative to the expression of GAPDH in aged mice treated with PTH or Veh for 1 or 2 months, respectively. (*n* = 5). Representative images and quantitative analysis of the length of PGP9.5-positive (**g**, **h**, Green) and CGRP-positive (**g**, **i**, Red) fibers in the vertebral body and endplate of WT young mice 2 months after LSI surgery and treated with PTH or Veh for 2 months. Scale bar: 100 µm. (*n* ≥ 6, *t*-test). Representative images (**j**) and quantitative mean IF intensity (**k**) of CGRP-positive neurons in the DRG of WT young mice 2 months after LSI surgery and treated with PTH or Veh for 2 months. Scale bar: 100 µm. (*n* ≥ 5, *t*-test). DAPI stains nuclei blue. EP endplate, VB vertebral body. *P < 0.05, **P < 0.01, ***P < 0.005, ****P < 0.001

To uncover the mechanism by which PTH treatment alleviates pain, we examined peripheral sensory innervation in the aged and LSI-mouse models, omitting the SM/J mice given their complex genetic background. Notably, we found a significant reduction in peripheral sensory nerve innervation as quantitated by length of PGP9.5 and CGRP-positive nerve fibers within the vertebral body and endplate in aged mice treated with PTH for 1 and 2 months, but not 2 weeks, relative to vehicle control (Fig. 2a–c and Fig. S2a, b). In-depth examination of CGRP expression in the first and second lumbar (L1/L2) dorsal root ganglion (DRG) showed a decrease in CGRP-positive neurons after 1 and 2 months of PTH administration in aged mice (Fig. 2d, e). Decreased protein levels of CGRP and PGP9.5 in DRG were further confirmed by Western blot in aged mice treated with PTH for 1 month compared with vehicle controls (Fig. 2f and Fig. S2c). Similar results were observed in young WT LSI mice treated with PTH relative to vehicle controls. Specifically, the relative length of PGP9.5-positive and CGRP-positive nerve fibers in the vertebral body and expression of CGRP in the L1/L2 DRG were significantly decreased in the WT LSI mice treated with PTH for 2 months relative to vehicle control (Fig. 2g–k). PTH treatment also reduced the expression of Isolectin IB_4_ (IB4) and Tyrosine hydroxylase (TH) in the spinal endplate (L5) relative to vehicle control in aged mice (Fig. S2d). Collectively, our findings suggest that PTH treatment relieved pain through decreased nociceptive innervation in spinal degeneration.

### Osteoblasts are primarily responsible for PTH-mediated spine rejuvenation

**Fig. 3:**
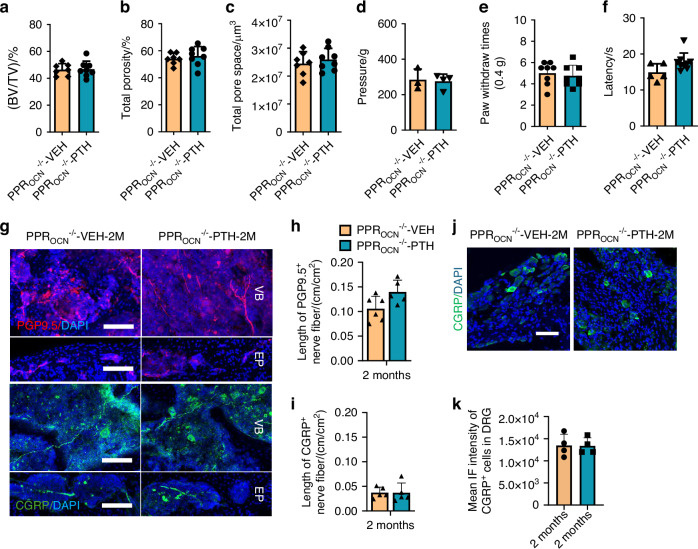
Loss of PPR in osteoblasts ameliorates PTH response in endplate degeneration and pain behaviors during spine degeneration. BV/TV percentage (**a**), total porosity percentage (**b**), and total pore space (**c**) in PPR_OCN_^−/−^ LSI mice with PTH or Veh treatment for 2 months. (*n* = 5, *t*-test). Behavior evaluation of PPR_OCN_^−/−^ LSI mice treated with PTH or Veh for 2 months included pressure tolerance of the lumbar spine region (**d**, *n* ≥ 3, *t*-test), paw withdraw times of the hind paw response to 0.4 g Von Frey filament stimulation (10 times in total) (**e**, *n* = 8, *t*-test), and latency of paw withdrawal post-thermal stimulation (**f**, *n* ≥ 7, *t*-test). Representative images of PGP9.5 and CGRP-positive fibers (**g**), quantitative analysis of fiber length in the lumbar vertebral body of PPR_OCN_^−/−^ LSI mice with PTH or Veh treatment (**h**, **i**). Scale bar: 100 µm. (*n* ≥ 5, *t*-test). Representative images showing CGRP-positive neuron (**j**) and quantitative analysis of mean intensity of immunofluorescence (**k**) in the DRG of PPR_OCN_^−/−^ LSI mice. Scale bar: 100 µm. (*n* = 4, *t*-test). *P < 0.05, **P < 0.01

We have previously published that the pain relief effects were not mediated through the IVD.^37^ To try to discern the primary cell type decreasing nociceptive innervation in response to PTH, we sought to selectively genetical delete PPR in osteoblasts using osteocalcin (OCN) Cre-drivers. Given that both aged and LSI-mice had similar endplate and behavior changes, we limited our genetic manipulation studies to the LSI-model. We knocked out PPR in the osteoblast lineage using OCN-Cre^39^ and performed LSI surgery (PPR_OCN_^−/−^ LSI mice). PPR was unaffected in the IVD, but nearly completely undetectable in OCN-positive cells within the endplate and trabecular bone of the vertebrate relative to wild-type mice; PPR was still noted in other cells within the endplate (Fig. S3a–d). Loss of PPR in OCN-lineage cells ameliorated the effects of PTH relative to vehicle. Specifically, there was no significant difference in BV/TV, total porosity, or total pore space between PTH treatment and vehicle control in PPR_OCN_^−/−^ LSI mice (Fig. 3a–c). Additionally, PTH no longer demonstrated efficacy in any of the behavior tests, although latency trended towards improvement (*P* = 0.051 5) (Fig. 3d–f and Fig. S3e). Delving deeper into the innervation patterns, despite the greater length of PGP9.5 nerve fibers relative to wild-type mice, we found no significant difference between the length of PGP9.5-positive and CGRP-positive nerve fibers in the vertebral bodies of PTH-treated PPR_OCN_^−/−^ LSI mice relative to vehicle-treated PPR_OCN_^−/−^ LSI mice (Fig. 3g–i). PTH treatment also did not significantly change the expression of CGRP in DRG neurons of PPR_OCN_^−/−^ LSI mice relative to vehicle (Fig. 3j, k). Altogether, our data suggests that OCN-lineage cells, namely osteoblasts and perhaps osteocytes are the principal cells responding to PTH treatment in regards to spinal hypersensitivity in our spine degeneration mouse models.

### Nerve repelling factors secreted by osteoblasts under PTH stimulation

**Fig. 4:**
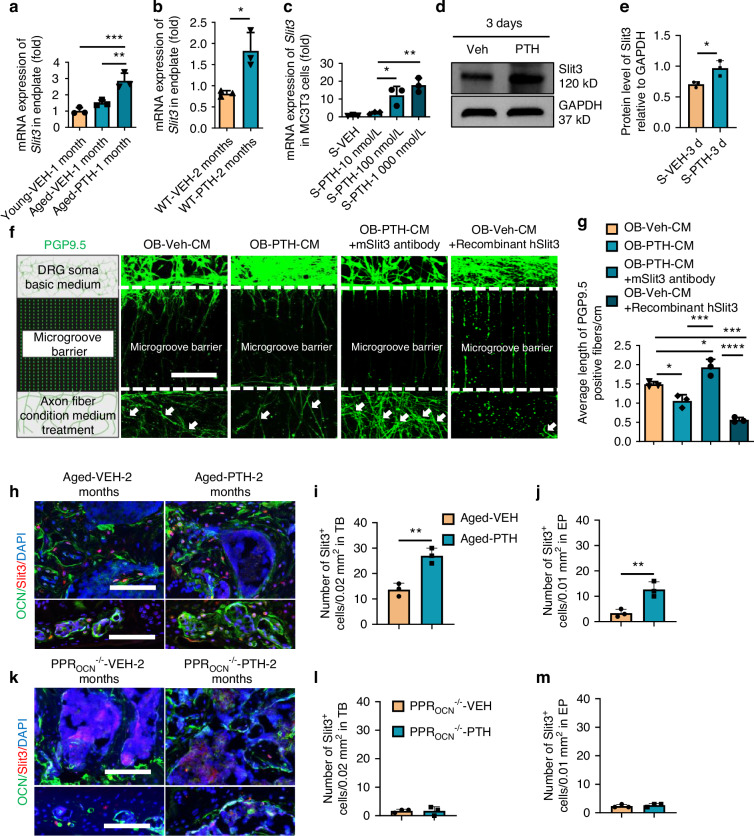
Osteoblasts respond to PTH treatment with increased Slit3 transcription and translation. mRNA expression of the *Slit3* in the endplate tissue of WT young mice treated with Veh and aged mice treated with Veh or PTH for 1 month (**a**), and WT LSI mice treated with veh or PTH for 2 months (**b**) (*n* = 3, one-way ANOVA with Tukey’s multiple comparisons test). **c** mRNA expression of the *Slit3* in MC3T3 cells cultured in osteoblast-stimulated medium and subsequently treated with Veh or PTH at different doses for 3 days (*n* = 3, *t*-test). **d**, **e** Protein expression level of Slit3 in MC3T3 cells cultured in osteoblast-stimulated medium and treated with Veh (PBS) or PTH for 3 days. (*n* = 3, *t*-test). Representative images (**f**) and quantification (**g**) of IF staining of PGP9.5 positive primary DRG neuron fibers crossing the microgroove barrier (microfluid assay) cultured in condition medium (CM), CM + PTH, CM + PTH plus mouse Slit3 antibody (1 µg/mL), or CM + Veh (1X PBS) plus human recombinant Slit3 (1.25 µg/mL) for 1 week. Representative images showing co-immunostaining for OCN (green) and Slit3 (red) in the lumbar spine sections and quantitative analysis of number of Slit3^+^ cells in trabecular bone (TB) and endplate (EP) of aged mice (**h**–**j**) and PPR_OCN_^−/−^ LSI mice (**k**–**m**) treated with PTH or Veh for 2 months. Scale bar: 100 µm. (*n* ≥ 5, *t*-test). *P < 0.05, **P < 0.01, ***P < 0.005, ****P < 0.001

Nerve fiber growth is directed by various factors, with semaphorins, ephrins, and Slits being the major nerve repellent factors.^40–42^ To identify the potential repulsive guidance factor responsive to PTH treatment, we first extracted total mRNA from the spine endplate of young and aged WT mice treated with either PTH or vehicle. Expression of the repellent factors genes *Slit3*, *Sema3a*, and *Efnb2* by quantitative polymerase chain reaction (qPCR) was significantly increased in PTH-treated aged mice relative to vehicle-treated aged mice; however, only *Slit3* in the PTH-treated aged mice was also significantly higher relative to young mice (Fig. 4a and Fig. S4a, b). Expression of *Slit3* was also significantly increased in PTH-treated WT-LSI mice relative to vehicle controls (Fig. 4b). To explore the mechanism of PTH-induced Slit3 secretion, we cultured the MC3T3 cell line in osteoblast-inducing medium (including 50 μg/mL ascorbic acid and 2 mmol/L of β-glycerophosphate) for 3 days. Gene expression by qPCR confirmed that the induced cells had significantly elevated expression of *Bglap, Col1a1, Sp7, and Runx2* genes relative to unstimulated controls (Fig. S4c–f), consistent with osteoblast differentiation. Stimulated MC3T3 cells were then cultured in vehicle or PTH at various concentrations in the presence of osteoblast-inducing medium for another 3 days; PTH significantly increased *Slit3* expression in a dose-dependent manner (Fig. 4c), whereas PTH inconsistently altered the gene transcription of *Sema3a* and *Efnb2*, increasing only at relatively lower concentrations with suppression at the highest PTH concentration (Fig. S4g, h). Slit3 protein concentration also significantly increased in PTH (100 nmol/L) treated MC3T3 cells relative to vehicle control (Fig. 4d, e). We also isolated primary osteoblast cells from WT mice, stimulated with osteoblast-inducing medium for 7 days, and treated with PTH (100 nmol/L) or vehicle for 3 days. PTH treatment significantly increased *Slit3* expression in primary osteoblasts relative to vehicle (Fig. S4i). To confirm nerve fiber repulsion by osteoblasts, we cultured primary DRG neurons and conducted a microfluidic assay using MC3T3 cell cultured condition medium with PTH or vehicle treatment, with or without Slit3 antibody treatment, as well as recombinant Slit3 as a positive control. The length of primary DRG nerve axons was significantly reduced in the PTH-treated-conditioned media and recombinant hSlit3-conditioned- media relative to the vehicle-treated-conditioned media. The PTH-treated-conditioned media with addition of the Slit3 antibody had significantly increased nerve fiber growth relative to vehicle-treated-conditioned media (Fig. 4f, g). Increased protein expression of Slit3 was confirmed in vivo by immunofluorescence staining in both aged and WT LSI mice treated with PTH for 2 months relative to vehicle control (Fig. 4h–j and Fig. S4j–l). There was no difference in Slit3 between PTH and vehicle treated groups in PPR_OCN_^−/−^ LSI mice (Fig. 4k–m). Overall, the results demonstrate that PTH treatment stimulated osteoblasts to secrete repellent factors, including Slit3.

### PTH stimulates Slit3 transcription in osteoblasts through FoxA2

**Fig. 5:**
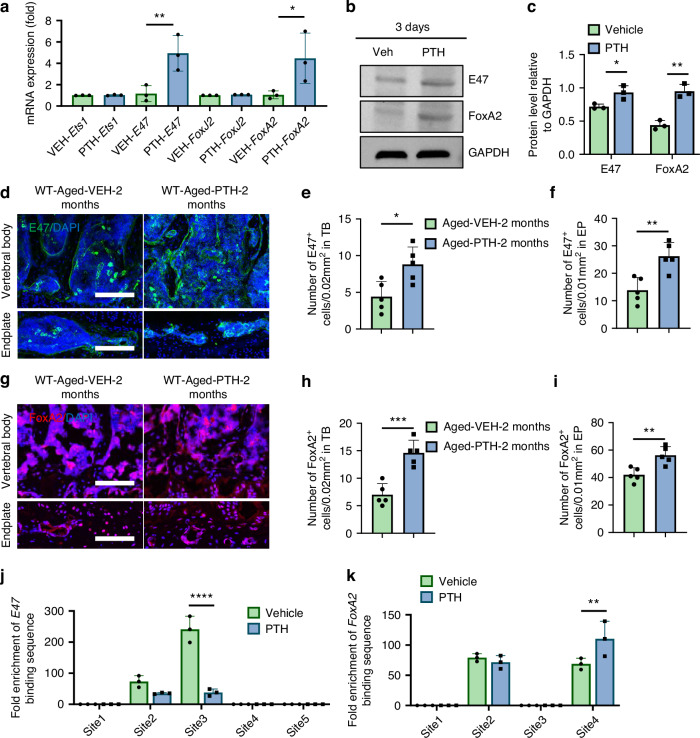
Transcriptional mechanism underpinning Slit3 secretion. **a** mRNA expression levels of *Ets1, E47, FoxJ2*, and *FoxA2* genes in MC3T3 cells cultured in osteoblast differentiation-inducing medium and treated with vehicle or PTH (100 nmol/L) for 3 days (*n* = 3, *t*-test). **b**, **c** Protein expression level of E47, FoxA2 in MC3T3 cells cultured in stimulated medium and treated with PTH or Veh (PBS) for 3 days (*n* = 3, *t*-test). Representative images depicting E47 IF staining (green) in lumbar vertebral body and endplate sections (**d**), quantitative analysis of the number of E47^+^ cells in the vertebral body (**e**) and endplate (**f**) of aged mice treated with Veh or PTH (*n* = 5, *t*-tset). Scale bar: 100 µm. Representative images showing FoxA2 IF staining (red) in lumbar vertebral body and endplate sections (**g**), quantitative analysis of the number of FoxA2^+^ cells in the vertebral body (**h**) and endplate (**i**) of aged mice treated with PTH or vehicle for 2 months (*n* = 5, *t*-tset). Scale bar: 100 µm. **j** Relative fold enrichment of the E47 binding site in the *Slit3* promoter region from stimulated MC3T3 cells treated with vehicle or PTH (100 nmol/L) for 3 days (*n* = 3, two-way ANOVA with Sidak’s multiple comparisons test). **k** Relative fold enrichment of the FoxA2 binding site in the *Slit3* promoter region from stimulated MC3T3 cells treated with vehicle or PTH (100 nmol/L) for 3 days (*n* = 3, two-way ANOVA with Sidak’s multiple comparisons test). *P < 0.05, **P < 0.01, ***P < 0.005, ****P < 0.001

Previous studies have indicated that the expression of *Slit3* can be regulated by transcription factors such as Ets1, E47, FoxJ2, and FoxA2.^43^ To elucidate how PTH modulates Slit3 transcription in osteoblasts, we cultured MC3T3 cells in osteoblast differentiation-inducing medium and treated with PTH (100 nmol/L) for another 3 days, followed by qPCR. Both *E47* and *FoxA2* mRNA expression were significantly increased in the PTH-treated group relative to the vehicle control (Fig. 5a). Using the same Western blots as shown in Fig. 4d, we found that the protein concentration of E47 and FoxA2 also significantly increased in PTH treated cells relative to vehicle control in MC3T3 cells (Fig. 5b, c). We then validated the expression of E47 and FoxA2 in the spine of aged mice. We observed that both E47 and FoxA2 were significantly upregulated in the spine endplate and vertebral body of aged mice treated with PTH for 2 months compared to those receiving vehicle (Fig. 5d–i). We performed the Chromatin Immunoprecipitation (ChIP) assay to confirm the transcriptional mechanism regulating *Slit3* gene expression. While both E47 and FoxA2 regulated transcription through two distinct binding sites located on the *Slit3* gene promoter region, only one binding site of FoxA2 exhibited a significant increase in transcriptional binding affinity upon PTH stimulation (Fig. 5j, k and Fig. 5a, b), suggesting that PTH treatment augments Slit3 secretion in osteoblast lineage cells primarily through FoxA2 transcriptional activation.

### Slit3 secreted by osteoblast lineage cells contributes to spine rejuvenation with PTH treatment

**Fig. 6:**
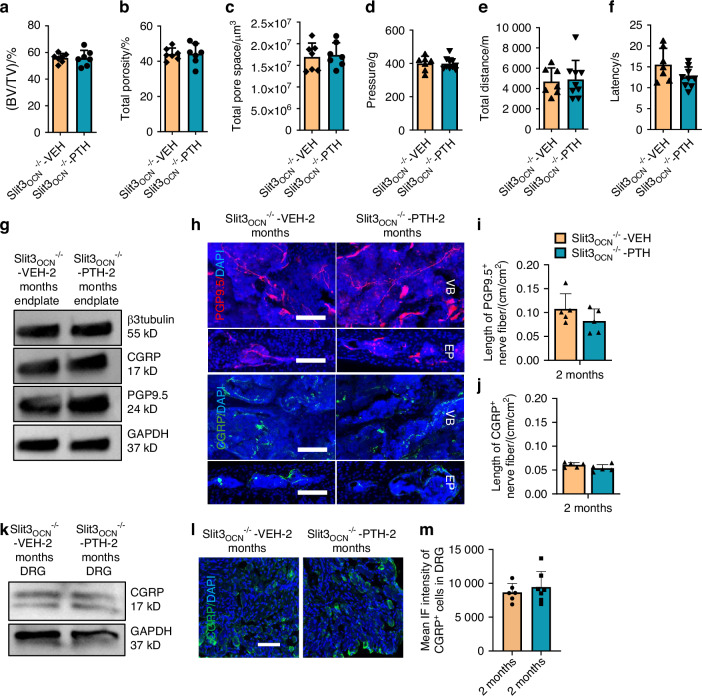
Efficacy of PTH treatment is diminished in osteoblastic Slit3 knockout mice with spinal degeneration. Vertebral endplate bone structure analyses by micro-CT: bone volume per tissue volume (BV/TV) percentage (**a**), total porosity percentage (**b**), and total pore space (**c**) in Slit3_OCN_^−/−^ LSI mice with PTH (40 µg/kg/d) or Veh treatment for 2 months (*n* = 7, *t*-test). Behavior evaluations included pressure tolerance of the lumbar spine assessed via the force threshold (**d**), total distance covered in spontaneous activity in 2 days (**e**), and latency of paw withdrawal post-thermal stimulation (**f**) for Slit3_OCN_^−/−^ LSI mice with PTH or Veh treatment for 2 months (*n* = 7, *t*-test). **g** Western blot analysis of protein expression levels of β3 tubulin, CGRP, PGP9.5 relative to GAPDH in endplate tissue (L3-L5) in Slit3_OCN_^−/−^ LSI mice treated with PTH or Veh for 2 months. (*n* = 5). Representative images of PGP9.5 (red) and CGRP (green)-positive fibers (**h**) and quantitative analysis of fiber length in the lumbar vertebral body of Slit3_OCN_^−/−^ LSI mice with PTH or Veh treatment (**i**, **j**) (*n* = 5, *t*-tset). Scale bar: 100 µm. **k** Western blot analysis of protein expression levels of CGRP in DRG tissue (L1–L2), normalized to GAPDH expression, in Slit3_OCN_^−/−^ LSI mice treated with PTH or Veh for 2 months (*n* = 5). Representative IF images showing CGRP-positive (green) neurons in DRG sections (**l**), followed by quantitative analysis of the mean IF intensity for CGRP (**m**) in the DRG of Slit3_OCN_^−/−^ LSI mice treated with PTH or Veh for 2 months (*n* = 5, *t*-tset). Scale bar: 100 µm

To confirm the significance of Slit3 secreted by osteoblast lineage cells in response to PTH treatment in spinal degeneration mice, we specifically knocked out the *Slit3* gene in osteocalcin-expressing cells, creating Slit3_OCN_^−/−^ mice. Slit3 was nearly completely undetectable, while PPR was unchanged in osteocalcin-positive cells within the endplate and trabecular bone of the vertebrate relative to wild type mice (Fig. 6a–f). Mice underwent LSI surgery at 2 months of age, observed for 2 months, then were injected with either PTH or vehicle for another 2 months. Micro-CT analysis indicated no significant difference in vertebral endplate BV/TV, total porosity, or pore size between the PTH-treated mice and the vehicle-treated groups (Fig. 6a–c). Importantly, deletion of *Slit3* in osteoblasts negated the pain-relieving efficacy of PTH treatment, as evidenced by lack of significant difference between PTH and vehicle groups on behavior tests (Fig. 6d–f). Furthermore, there was no differences in the expression levels of β3 tubulin, PGP9.5 and CGRP in L5 endplate extracted protein in Slit3_OCN_^−/−^ PTH-treated LSI mice relative to and Slit3_OCN_^−/−^ Vehicle-treated LSI controls (Fig. 6g). There was also no significant difference of the peripheral sensory nerve fibers in the vertebral body or endplates between PTH and vehicle treated groups (Fig. 6h–j). Finally, neither the protein concentration nor the mean fluorescence intensity of CGRP in the L1/L2 DRG tissue demonstrated significant difference between PTH-treated Slit3_OCN_^−/−^ LSI mice and the vehicle-treated control group (Fig. 6k–m). Altogether, depletion of *Slit3* in osteoblast lineage cells eliminated the efficacy of PTH treatment in spinal degeneration mice.

## Discussion

LBP is a prevalent clinical problem with a series of complex etiologies based on the anatomy of the spine, including spinal stenosis, facet arthropathy, myofascial pain, IVD degeneration, herniated nucleus pulposus, and endplate degeneration.^2,44^ We examined three mouse models with spinal hypersensitivity due to either spinal degeneration or instability and described a unifying phenotype regarding LBP. The LSI surgery and aged models have been reported as unique LBP mouse models for in vivo study.^12,13,45^ The complex transgenic mouse strain, SM/J, develops IVD degeneration spontaneously and earlier, severe by 4–8 weeks of age, due to multiple genetic variants within its background.^38,46^ We previously demonstrated that PTH treatment significantly improved spine endplate and IVD degeneration and pain in both the LSI surgery model and aged models by reducing local nociceptive innervation.^37^ In the current study, we validated spinal rejuvenation with PTH treatment in WT aged, LSI, and SM/J models, and further characterized the dynamic pathological characteristics of aged and LSI-induced LBP in both bone structure and nociceptive innervation; the SM/J mice contain a complex genetic background which could influence the mechanism. Most importantly, we demonstrated that PTH orchestrates nociceptive axon repulsion in the vertebral body and endplate by enhancing *Slit3* transcription in osteoblast-lineage cells, decreasing aberrant sensory innervation and alleviating pain. PTH treatment also significantly decreased expression of CGRP and PGP9.5 within the DRG of both aged and LSI mouse models, underscoring the potential of PTH treatment in addressing the neuropathic components of LBP during spinal degeneration.

LBP may arise from uncoupled bone remodeling with an imbalance of osteoclast and osteoblast activity in the vertebral endplate and body. Initially, a young, healthy endplate comprises chondrocytes embedded in a collagen matrix. With aging and degeneration, chondrocytes undergo hypertrophy and ossification, leading to the formation of marrow-filled pores.^47,48^ Both osteoclasts and osteoblasts are involved in pore formation and metabolic activities during endplate degeneration. Bone homeostasis is regulated through osteoclast resorption and osteoblast formation, mediated by cytokines such as TGF-β and IGF-1.^49,50^ Overactivity of osteoclasts can disrupt this balance, leading to uncoupling and pain in degenerative diseases like osteoarthritis and LBP. We have previously shown that Netrin-1, secreted by osteoclasts, acts as a key nerve axon attractant factor, increasing nociceptive sensory innervation to the affected regions as observed in models of osteoarthritis and LBP.^13,28^ This study highlights that the nerve repellant factor, Slit3, is increased in response to PTH, counteracting the overactivity of osteoclasts and facilitating sensory denervation to mitigate LBP.

The mechanism of nociceptive denervation is multifaceted and includes Slit3, Sema3a, and EphrinB2 as the major repellant guidance factors. We found that Slit3 was significantly increased in PTH-treated aged mice relative to both vehicle-treated aged and younger mice. In contrast, the expression levels of Sema3a and EphrinB2 did not significantly differ between young and aged mice. In vitro experiments revealed that high doses of PTH suppressed the expression of Sema3a and EphrinB2. We identified that OCN-lineage cells mediate PTH-stimulated Slit3 production. Using osteoblast cells lines and isolation/differentiation from bone marrow, we identified PTH could increase Slit3 and two of its transcription factors, FoxA2 and E47, both in vitro and in vivo. However, only the binding affinity of FoxA2 was increased in response to PTH. These results suggest that while the transcriptional factor E47 may stimulate *Slit3* transcription, E47 does not appear to be regulated by PTH treatment. The increased expression of E47 in the spine section could instead be regulated by other cell types that respond to PTH treatment or may be increased by non-PTH pathways.

The therapeutic efficacy of PTH in enhancing bone formation in osteoporosis is well-documented, and the underlying mechanisms have been extensively studied.^29,31^ Our findings suggest a mechanistic role of PTH in bone pain modification, particularly in degenerative joint conditions in animal models of osteoarthritis and LBP.^37,51^ Potential analgesic effects of PTH have also been reported in human clinical trials, such as teriparatide and abaloparatide, synthetic analogs of human PTH and PTH-related peptide, respectively, where improvements in LBP were reported following treatment,^4,52–54^ although not always consistently.^55^ Most of the studies were not designed to study pain specifically but were often collected as an adverse event. One study reported and equal complaint of pain between teriparatide, abaloparatide, and placebo groups.^55^ We also note that the inclusion criteria of these studies focused on osteopenia/osteoporosis and did not stratify by pathological changes of vertebral endplates which may be a critical criterion for future clinical trials for use in spinal degeneration.

Our study begins to elucidate potential mechanisms through which PTH alleviates pain. Our research posits that Slit3 acts as a critical nerve repellant factor, playing a significant role in mitigating pain by reducing aberrant innervation is response to PTH treatment in spinal degeneration. Intriguingly, Slit3 has also been identified as a factor promoting bone formation, secreted by osteoclasts^56^ and as a proangiogenic factor essential for the CD31^hi^EMCN^hi^ blood vessels^,^ secreted by osteoblasts^57^; its absence leads to reduced bone mass.^58^ Both Slit3 and its receptor, Robo1, are implicated in bone metabolism and the maintenance of skeletal homeostasis.^59,60^ Similar to prior reports,^58^ we found that loss of Slit3 in osteoblast lineage cells significantly reduced the bone volume of the vertebral body. However, interestingly loss of osteoblast Slit3 did not inhibit the increase of bone volume in the endplate noted during spinal degeneration of the LSI mice (Fig. S6g–k). This differing effect of bone volume based on location suggests a unique mechanism in the endplate relative to the vertebral body, which should be investigated further in future studies, particularly in regards to changes in bone vasculature. Our findings suggest that PTH-induced osteoblast-transcription of Slit3 also plays a role in neuronal guidance, decreasing nociceptor innervation and providing pain relief. The multifaceted role of Slit3 in bone formation, angiogenesis, and innervation suggests that Slit3 may be a promising therapeutic target for addressing bone degeneration issues, offering benefits from both skeletal and neuropathic perspectives.

There are limitations in our study that should be addressed in future studies. We quantified the sensory innervation by measuring the length of the PGP9.5 and CGRP positive fibers in the spine from each 40 μm slice. The relative length was calculated by dividing the length by the area of the spine tissue, which could result in missing some fibers. The relative length of PGP9.5 fibers was significantly increased in PPR_OCN_^−/−^ mice compared to WT control, but we did not conduct additional experiments to explore this mechanism aside from presumed decreased endogenous PTH signaling. We observed that PTH treatment may influence interoception signaling by reducing IB4-positive nociceptive sensory fibers and TH-positive sympathetic nerve fibers in the vertebral endplate. The reduction in IB4 expression suggests a decrease in nociceptive input, which could attenuate abnormal interoceptive-driven bone formation at the endplate. Additionally, the reduction in TH expression may reflect a decrease in sympathetic tone, which is known to influence bone remodeling. The precise mechanisms underlying these observations remain to be explored in future studies. Osteocalcin may be expressed in other organs throughout the body, such as the hypothalamus or hippocampus; loss of PPR in these regions was not evaluated as a potential confounder for the observed effects.^61,62^ Consequently, we acknowledge the possibility that central nervous system effects may influence the overall response to PTH treatment, potentially affecting both bone remodeling and pain perception outcomes. Based on the literature, there may also be differences of spinal innervation based on genetic background as the osteoblast lineage highly crosstalks to the nervous system through Runx2, Sema, Wnt4, Shh, NGF, BDNF, etc.^63–67^ Our study focused on the mechanism of the PTH-stimulated osteoblast Slit3 in regards to pain and aberrant skeletal innervation. Slit3 has important roles in bone formation directly through osteoblasts and indirectly through type H vessels. Future studies are needed to understand if these processes are affected by PTH during spinal degeneration.

Overall, our study elucidates a downstream mechanism of PTH attenuation of LBP in spinal degeneration mouse models, providing further insight how bone remodeling modulates the catabolic and anabolic balance between osteoclasts and osteoblasts to preserve bone homeostasis and regulate nociceptive innervation. Combined with prior studies, the pain signal in the degenerated endplate is transmitted by nociceptive nerve fibers, while the nociceptive innervation is regulated by neuronal guidance factors, such as the attractant factor Netrin-1 and the repellant factor Slit3, which are predominantly secreted by osteoclasts and osteoblasts, respectively.^13^ Abnormal bone coupling was triggered during mechanically-induced spinal degeneration as well as aging, furthering aberrant innervation coupled to osteoclast activity. The excessive osteoclasts result in the secretion of Netrin-1, that could trigger peripheral sensitization for nociceptive pain by attracting nerve fiber growth. In our study, PTH signaling via osteoblast lineage cells resulted in increased Slit3 to decrease the nociceptive fibers and mitigate pain, while also restoring coupled bone remodeling (Fig. S7a). Overall, PTH treatment of LBP during spinal degeneration appears to be related to restoring coupled bone remodeling and reducing aberrant innervation, providing proof-of-concept for future clinical trials exploring the efficacy of PTH as a disease-modifying and pain-relief treatment for spinal degeneration.

## Materials and methods

### Animals models

The study utilized various mouse genotypes, including C57BL/6J (WT), SM/J, PPR_Col2a_^ERT2−/−^, PPR_OCN_^−/−^, and Slit3_OCN_^−/−^. WT young mice (#000664, 2–3 months of age) and SM/J mice (#000687) were purchased from the Jackson Laboratory in USA. WT aged mice (22 months of age) were obtained from the National Institute on Aging in USA. The *Pth1r*(PPR) flox/flox mice were obtained from H. Kronenberg at Massachusetts General Hospital, located in Boston, MA, USA. The *Osteocalcin*(OCN)-Cre mouse line was contributed by Thomas L. Clemens at Johns Hopkins University, located in Baltimore, Maryland, USA. We also acquired the *Slit3* flox/flox mouse line from Jung-Min Koh at University of Ulsan College of Medicine, located in Songpa-Gu, Korea. To confirm genotypes, we performed polymerase chain reaction (PCR) analysis. This analysis involved extracting genomic DNA from the tails of the mice and utilizing a set of specific primers.

*Pth1r* Forward: 5′- TGGACGCAGACGATGTCTTTACCA -3′,

*Pth1r* Reverse: 5′- ACATGGCCATGCCTGGGTCTGAGA -3′;

*Osteocalcin* Transgene Forward: 5′- TCCTCAAAGATGCTCATTAG -3′,

*Osteocalcin* Transgene Reverse: 5′- GTAACTCACTCATGCAAAGT -3′,

*Osteocalcin* Internal positive control Forward: 5′- CAAATAGCCCTGGCAGAT -3′,

*Osteocalcin* Internal positive control Reverse: 5′- TGATACAAGGGACATCTTCC -3′;

*Slit3* Forward: 5′-GATTCTAAGAGCCTGCTTAG -3′,

*Slit3* Reverse: 5′-GACACTGGAGCGTAGGACTCC -3′.

In this study, Lumbar Spine Instability (LSI) surgery was conducted on adult male mice aged between 2 to 3 months. The mice groups included WT, PPR_OCN_^−/−^, and Slit3_OCN_^−/−^ genotypes. Anesthesia was induced by administering ketamine 100 mg/kg and xylazine 10 mg/kg intraperitoneally. For the LSI model, the L3–L5 spinous processes and the supraspinous and interspinous ligaments were surgically removed. For the sham procedure, only the posterior paravertebral muscles from the L3–L5 vertebrae were detached, without affecting the spine’s stability. Post-surgery, all mice were housed and cared for at the animal facility of The Johns Hopkins University School of Medicine. PTH (1-34, H-4835.0005, Bachem) treatment was intraperitoneally administered (40 µg/kg/d) for 2 weeks, 1 month, or 2 months. The animal protocol was approved by the Institutional Animal Care and Use Committee of Johns Hopkins University, Baltimore, MD, USA.

### Micro CT

The mice in the study were humanely euthanized through an overdose of isoflurane, followed by perfusion with 1X Phosphate-Buffered Saline (PBS) and 10% buffered formalin. For evaluating the endplates, L5 segments of the lumbar spine were extracted and subjected to micro-Computed Tomography (μCT) analysis. The μCT parameters included a voltage of 55 kVp, a current of 181 μA, and a resolution of 9.0 μm per pixel, using a Skyscan 1172 system. The μCT images were processed using the NRecon v1.6 software (Skyscan) for reconstruction. Quantitative assessments of these images were carried out using the CTAn v1.9 software (Skyscan). Regarding the endplates, we chose six consecutive images of the caudal endplates of L4-L5 and the L5 vertebrae in the coronal view. These images were utilized for 3D reconstruction using the CTVol v2.0 software (Skyscan).

### Pressure tolerance test

All behavioral assessments were conducted by an investigator who was blinded to the groupings of the mice. We utilized the SMALGO algometer (Bioseb) to measure pressure thresholds, which served as an indicator of pressure hyperalgesia. During the procedure, a sensor tip with a diameter of 5 mm was applied to the L4-L5 spinal region of each mouse while the mice were under gentle restraint. The pressure was incrementally increased at a rate of 50 grams per second until the mouse emitted a vocalization, indicating the threshold of pressure tolerance. The pressure force was recorded using the BIO-CIS software (Bioseb), with a maximum limit set at 500 grams to avoid causing tissue damage. Between each testing session, the mice were given a 15-min rest/recovery period. The average of these measurements was then calculated to determine the final pressure tolerance threshold for each mouse.

### Active wheel test

For the assessment of spontaneous wheel-running activity, we employed specialized mouse activity wheels (BIO-ACTIVW-M model, Bioseb). This setup included software capable of tracking and recording each mouse’s activity levels within the wheel cage. Prior to the commencement of testing, mice were allowed an overnight period to acclimate to the wheel cage environment. During the testing period, the mice experienced a 12-h light/dark cycle. Each mouse was monitored for a continuous period of 48 h. Software automatically logged various parameters pertaining to movement of the activity wheel.

### Hargreaves test

The Hargreaves method was employed to evaluate analgesia levels in various groups of mice. Each group of mice was first given an hour to become accustomed to the testing environment. For the test, a focused beam of radiant heat (provided by IITC Life Science Inc.) was directed onto the plantar surface of the hind paws of the mice. The response time, assessed as the time duration until the mouse withdrew its paw, was carefully measured. This response time, indicative of the latency period to the heat stimulus, was recorded for each paw. To ensure accuracy and consistency, the procedure was repeated a minimum of five times per mouse. The average of these latency times was then calculated and used for subsequent analysis.

### Von Frey test

To evaluate mechanical nociception in mice, we employed a Von Frey filament-based approach. Mice were individually placed on a raised metal grid enclosed within transparent plastic chambers and allowed to habituate for a minimum of 1 h prior to testing. A calibrated monofilament exerting 0.4 g of force (BIOSEB) was applied to the plantar surface of each hind paw. The stimulus was delivered for up to 3 s or until a withdrawal response occurred. Responses included paw retraction, flinching, or licking. Each hind paw was stimulated ten times with an inter-stimulus interval of two seconds. The frequency of paw withdrawals was recorded and expressed as a percentage of total trials. Each animal underwent the test on at least three separate occasions, and the mean withdrawal frequency was computed for analysis.

### Immunofluorescence staining

Upon euthanasia, bone specimens, specifically the L3-L5 lumbar spine, were harvested and immediately fixed in 10% buffered formalin for a duration of 24 h. The L1-L2 DRG tissues were isolated and fixed in 10% buffered formalin overnight. Subsequently, the bone samples underwent decalcification in 0.5 mol/L ethylenediaminetetraacetic acid at a temperature of 4 °C for 3 weeks, accompanied by constant agitation. The samples were embedded in optimal cutting temperature embedding medium (Sakura).

For histological examination, we prepared 40 μm thick spine or 10 μm thick DRG tissue sections for immunofluorescence staining following our previously reported protocol.^68^ The spine sections were incubated for 48 h at 4 °C with primary antibodies targeting CGRP (1:100, ab81887, Abcam), PGP9.5 (1:200, SAB4503057, Sigma), incubated overnight at 4 °C with primary antibodies targeting Osteocalcin (1:200, M188, Takara), PTH1R (1:100, ab75150, Abcam), Slit3 (1:100, AF3629, Biotechne), FoxA2 (1:100, 22474-1-AP, Proteintech), and E47 (1:100, sc-416, Santa Cruz), while the DRG sections were incubated overnight at 4 °C with primary antibodies targeting CGRP (1:100, ab81887, Abcam) and β3 tubulin (1:100, 2G10, Thermo Fisher). These were followed by the application of appropriate secondary antibodies and DAPI (1:250, H-1200, Vector) for 1 h in a light-protected environment. For the visualization and documentation of the samples, we employed both a fluorescence microscope (Olympus BX51, DP71) and a confocal microscope (Zeiss LSM 880). Quantitative analyses of the images were performed using ImageJ software (National Institutes of Health, Bethesda, MD).

### Western blot

We pulverized the endplate tissue samples in a liquid nitrogen environment to facilitate the extraction of total protein. This extraction was carried out using the T-PER™ Tissue Protein Extraction Reagent (catalog number 78510, Thermo Fisher), complemented with 1% Protease and Phosphatase Inhibitor cocktail (catalog number 78442, Thermo Fisher). For cell culture lysates, we utilized RIPA buffer (catalog number 89901, Thermo Fisher), also supplemented with 1% of the aforementioned cocktail. Lysates obtained were then centrifuged and their protein concentrations standardized using the BCA Protein Assay Kit (catalog number 23227, Thermo Fisher).

The protein samples prepared were subsequently resolved by electrophoresis on a 10% SDS-PAGE gel and transferred to polyvinylidene difluoride membranes (sourced from Bio-Rad Laboratories). The membranes, post-transfer, were blocked with 5% fat-free milk and incubated overnight with specific primary antibodies at 4 °C. Following this, membranes were washed with Tris-buffered saline mixed with 0.05% Tween-20 and incubated with horseradish peroxidase-conjugated secondary antibodies.

For protein detection, we utilized an enhanced chemiluminescence kit (Thermo Fisher Scientific). A range of primary antibodies was used for this purpose, including those specific for mouse β3 tubulin (1:500, 2G10, Thermo Fisher), CGRP (1:1 000, sc-57053, Santa Cruz), PGP9.5 (1:1 000, SAB4503057, Sigma), IB4 (1:1 000, I21441, Thermo Fisher), TH (1:1 000, AB152, Sigma), Slit3 (1:1 000, AF3629, Biotechne), E47 (1:1 000, sc-416, Santa Cruz), FoxA2 (1:1 000, 22474-1-AP, Proteintech), and GAPDH (1:2 000, 14C10, Cell Signaling), which facilitated the determination of protein concentrations in the lysates.

### MC3T3 cell culture

MC3T3 subclone 4 cell line was purchased from ATCC (CRL-2593^TM^) and cultured using Alpha Minimum Essential Medium with ribonucleosides, deoxyribonucleosides, 2 mmol/L L-glutamine and 1 mmol/L sodium pyruvate, but without ascorbic acid (A10490-01, Thermo Fisher). The osteoblast differentiation medium was supplied with 50 μg/mL ascorbic acid (Sigma) and 2 mmol/L of β-glycerophosphate (G9422, Sigma) to induce osteoblast differentiation for 3 days. PTH (1-34) was diluted in 1X PBS into different doses for cell treatment. Cells were cultured with 10% fetal bovine serum (35-011-CV, Sigma-Aldrich) at 37 °C in a 5% CO_2_-humidified incubator.

### Primary osteoblast cell isolation and treatment

We followed the protocol reported by Chevalier.^69^ Clean bone was collected by flushing bone marrow from the femur and tibia of young WT mice. Cut bone pieces were digested in type1 collagenase solution (1 mg/mL) for 90 min at 37 °C in the cell incubator. The digested bone pieces were cultured in alpha MEM medium for 3 days, and refreshed every other day until the cells reached 80% confluence for passaging. The third generation of cells were seeded in 6-well plates for the treatment. Cells were stimulated in osteoblast differentiation inducing medium for 7 days, followed by PTH (100 nmol/L) or PBS treatment for 3 days. The total mRNA samples were collected for the qPCR experiments.

### qPCR test

Total RNA was extracted from the spine endplate tissue or cultured cells using RNeasy Plus Mini Kit (74134, Qiagen) according to the manufacturer’s instructions. The purity of RNA was tested by measuring the ratio of absorbance at 260 nm over 280 nm. For RT-PCR, 500 ng of RNA was reverse transcribed into complementary DNA using the PrimeScript™ RT Master Mix (RR036A, Takara), then RT-PCR was performed with SYBR Green-Master Mix (Qiagen) using QuantStudio 3 Real-Time PCR System (Thermo Fisher). Relative expression was calculated for each gene by the 2^-ΔΔct^ method, with glyceraldehyde 3-phosphate dehydrogenase (*Gapdh*) for normalization as reported.^70^ Primers used for RT-PCR were:

*Slit3* Forward: 5′- TGCCCCACCAAGTGTACCT -3′,

*Slit3* Reverse: 5′- CGCCTCTCTCGATGATGCT -3′;

*Sema3a* Forward: 5′- CACTGGGATTGCCTGTCTTTT -3′,

*Sema3a* Reverse: 5′- TGGCACATTGTTCTTTCCGTTT -3′;

*Efnb2* Forward: 5′- GCTAGAAGCTGGTACAAATGGG -3′,

*Efnb2* Reverse: 5′- CATCGGTGCTAGAACCTGGA -3′;

*Bglap* Forward: 5′- CTGACCTCACAGATCCCAAGC -3′,

*Bglap* Reverse: 5′- TGGTCTGATAGCTCGTCACAAG -3′;

*Col1a1* Forward: 5′- GCTCCTCTTAGGGGCCACT -3′,

*Col1a1* Reverse: 5′- CCACGTCTCACCATTGGGG -3′;

*Sp7* Forward: 5′- ATGGCGTCCTCTCTGCTTG -3′,

*Sp7* Reverse: 5′- TGAAAGGTCAGCGTATGGCTT -3′;

*Runx2* Forward: 5′- ATGCTTCATTCGCCTCACAAA -3′,

*Runx2* Reverse: 5′- GCACTCACTGACTCGGTTGG -3′;

*Ets1* Forward: 5′- TCCTATCAGCTCGGAAGAACTC -3′,

*Ets1* Reverse: 5′- TCTTGCTTGATGGCAAAGTAGTC -3′;

*E47* Forward: 5′- GGGTGCCAGCGAGATCAAG -3′,

*E47* Reverse: 5′- ATGAGCAGTTTGGTCTGCGG -3′;

*FoxJ2* Forward: 5′- GCCTCCGACCTGGAGAGTAG -3′,

*FoxJ2* Reverse: 5′- CTGTACCGTGGCTTGCCAT -3′;

*FoxA2* Forward: 5′- CCCTACGCCAACATGAACTCG -3′,

*FoxA2* Reverse: 5′- GTTCTGCCGGTAGAAAGGGA -3′;

*Gapdh* Forward: 5′- CATCACTGCCACCCAGAAGACTG-3′,

*Gapdh* Reverse: 5′- ATGCCAGTGAGCTTCCCGTTCAG-3′.

The primers were obtained from the Primer Bank Database, the specificity and linearity were verified and reported by others. We did the BLST to ensure specificity for the target gene with no significant off-target binding, further confirmed by a single sharp peak from the Melt Curve. Linearity was assessed by constructing a standard curve using serial 10-fold dilutions of the template.

### Primary DRG neuron isolation and culture

Young WT mice were euthanized as described above for DRG tissue isolation. We dissected the DRG tissue from thoracic and lumbar vertebrate under microscope and collected in F12 Minimum Essential Medium (F12-MEM, Gibco) supplemented with 1% Penicillin-Streptomycin solution (P.S.) at 4 °C. The medium was then replaced by 1 mL collagenase Type I solution (1 mg/mL, 17100017, Gibco) and incubated in a microcentrifuge tube at 37 °C for 90 min. Collagenase solution was then replaced with 500 μL 1X TrypLE™ Express Enzyme solution (12604013, Gibco) and incubated at 37 °C for 15 min. The specimen was centrifuged and the tissue pellet was collected (1 000 r/min, 5 min). The pellet was resuspended using F12-MEM medium containing 1X supplement B27 (17504044, Gibco) and filtered using 40 μm strainer. Prior to use in experiments, the DRG neurons were collected by centrifuge under 1 000 r/min for 5 min.

### Microfluid assay

For our neuron culture studies, we employed the Innsbruck Neuron Device (IND500) featuring a 500-µm microgroove barrier. This device was set up on a Corning cell culture dish with a 10 cm diameter. Initially, the device underwent a cleaning process involving an overnight soak in 10% hydrochloric acid, followed by a thorough ultrasonic cleaning in distilled and deionized water, repeated three times for 20 min each session. Prior to each experimental run, the device was air-dried and placed onto the culture dish. The dish wells were prepared by applying 100 μL of a coating solution that contained 100 μg/mL Poly-D-Lysine for 1 h at 37 °C, then coated with 10 μg/mL Laminin to each well after 1X PBS washing five times. The plate was incubated at 37 °C for 1 h, then the coating solution was discarded, and the wells were rinsed thrice with sterile 1X PBS.

DRG neurons were introduced into the central channel of the device. The successful migration of neurons into the designated channel was confirmed via microscopy. Subsequently, about 150 µL of culture medium was dispensed into each side well and cultured for 3 days before further intervention. Different interventions were administered to the wells: 150 µL conditioned medium from vehicle or PTH-treated osteoblasts, with or without Slit3 antibody (1 µg/mL, AF3629, R&D Systems), or human recombinant Slit3 protein (1.25 µg/mL, 9067-SL, Biotechne), for 1 week. Nerve growth factor (50 ng/mL, N-100, Alomone Labs) was supplemented for each well. After 1 week of incubation, the neurons and their axons were fixed and prepared for immunofluorescence staining.

For staining, the culture medium was removed, and cells were fixed using 4% paraformaldehyde (PFA, 200 μL/well) for 15 min at room temperature. Following fixation, the cells underwent three 1X PBS washes and were blocked with a solution containing 1% bovine serum albumin, 0.3% Triton X-100, and 2% normal goat serum in 1X PBS for an hour at room temperature. Axons were labeled with PGP9.5 antibody (1:200, SAB4503057, Sigma) and incubated overnight at 4 °C. Post-secondary antibody treatment, the wells were washed and prepared for confocal microscopy analysis using a Zeiss LSM 880 system.

### Chip assay

MC3T3 cells were cultured with osteoblast differentiation medium for 3 days and treated with PTH (100 nmol/L) or vehicle for another 3 days. Chip assay was performed according to the manufacturer’s protocol (Pierce™ Agarose CHIP Kit, Cat. 26156, Thermo Fisher). Briefly, we crosslinked the cell pellet using Glycine Solution after fixation in 1% formaldehyde. The cells were lysed in membrane extraction lysis buffer and nuclear extraction lysis buffer, along with MNase digestion (DTT, MNase Digestion Buffer). Of the sample, 10% was removed as an input control. Antibodies targeting E47 (sc-416, Santa Cruz), FoxA2 (22474-1-AP, Proteintech) were utilized. Additionally, anti-RNA polymerase II and control IgG served as the positive and negative controls, respectively. The DNA samples were further analyzed by qPCR and electrophoresis as introduced by the manufacturer. PCR primers used to detect E47 and FoxA2 binding site were as follows:

E47 Site #1: Forward: 5′- TCAGCCCTGGTACTAAAT -3′,

Reverse: 5′- CAAACCTTGAACCAATTT -3′;

E47 Site #2, Forward: 5′- GAGGACTGAGGCAAAGGC -3′,

Reverse: 5′- CTCTGCTTCCGATGGTGA -3′;

E47 Site #3, Forward: 5′- AGGCTATTTCAGACCTTT -3′,

Reverse: 5′- CAGGCTCCATACATACTTG -3′.

E47 Site #4, Forward: 5′- AGAACGGTGGCACCTTGA -3′,

Reverse: 5′- GCGGACCTTTATTTCCTTATTT -3′.

E47 Site #5, Forward: 5′- CCTACAGGCTCTTGGTTGCTC -3′,

Reverse: 5′- CGCTCGCTTTCTCCATTCAC -3′.

FoxA2 Site #1: Forward: 5′- TGGGGGTGGGGGGGGGGAGCTGGGG -3′,

Reverse: 5′- TCTTCTATTTTCCTTAAAGGAAACT -3′;

FoxA2 Site #2, Forward: 5′- TCAAGGAAGTCTGGGCAATA -3′,

Reverse: 5′- GGCAGGAACTGGAGGAAA -3′;

FoxA2 Site #3, Forward: 5′- TAGTTGTTGGCCTTAGCT -3′,

Reverse: 5′- TGAAATGATTATCCGAGAC -3′.

FoxA2 Site #4, Forward: 5′- GGGAGGCGGAGCTGGTGTTT -3′,

Reverse: 5′- GCGCTCGCTTTCTCCATTCAC -3′.

### Statistics

Statistical evaluations were conducted utilizing GraphPad Prism version 8.0 (Boston, MA, USA), with outcomes expressed as the mean ± standard deviation. Differences among multiple experimental groups were assessed using one-way Analysis of Variance (ANOVA) followed by Tukey’s multiple comparison test. Comparisons between two distinct groups were using an unpaired, two-tailed Student’s *t* test. A *p* value of less than 0.05 was designated as the threshold for statistical significance across all experimental conditions.

## Supplementary information

Supplementary Figures and legends

supplementary figure 1

supplementary figure 2

supplementary figure 3

supplementary figure 4

supplementary figure 5

supplementary figure 6

supplementary figure 7

original bands of WB in Figure 4d and Figure 5b

## Data Availability

All data associated with this study are present in the paper. Access to any additional information in this study is available upon request from W.Z. (wzhan108@jh.edu) and J.C. (jcrane2@jhmi.edu).
