# Supplementary material for: PTH induced osteoblast Slit3 to decrease aberrant sensory innervation in degenerated vertebral endplates to relieve low back pain in mice
Source: Bone Res. 2026 Jan 22;14:12. doi: 10.1038/s41413-025-00488-z (PMC12827982; doi:10.1038/s41413-025-00488-z)
Supplement: Supplementary file 1 — Supplementary Figures and legends [file 41413_2025_488_MOESM1_ESM.docx]

**Supplementary information**

**Supplementary Figure 1. Bone structure and pain behavior of three spinal degeneration mouse models. (a)** Schematic diagrams show the lumbar spine instability (LSI) surgery, time points of surgery, parathyroid hormone (PTH)/Vehicle (Veh) administration and sample collection of lumbar spine instability (LSI) surgery, aged, and SM/J mice. Black arrow: Sample collection timepoints. **(b-d)** Vertebral endplate bone structure analyses by micro-computed tomography (μCT): Bone volume/Tissue volume (BV/TV) (b), Total porosity percentage (c) and Total pore space (d) of L5 endplate of aged mice treated with PTH for two weeks relative to Veh-treated group as assessed by three-dimensional reconstruction of μCT. (n ≥ 5, *t*-test). **(e-g)** Representative μCT of the L5 vertebral endplate from aged mice (e), young WT mice post-LSI surgery (f), and six-month-old SM/J mice (g) treated with PTH or Veh for one or two months as indicated. Scale bar: 1mm. **(h-i)** Behavior evaluations included pressure tolerance in the lumbar spine region (h) and latency of hind paw withdrawal post-thermal stimulation (i) in aged mice treated with PTH or Veh for two weeks. (n ≥ 8, *t*-test). **P*＜0.05.

**Supplementary Figure 2. Analysis of Different Nerve Fiber Subtypes in Degenerated Spine Following PTH Treatment. (a-b)** Representative images (a) and quantitative analysis (b) of PGP9.5-positive fiber length in the lumbar vertebral body and endplate of aged mice treated with PTH or Veh for two weeks. Scale bar: 100 µm. (n = 7, *t*-test). **(c-d)** Relative protein levels of PGP9.5 (c), IB4 and TH (d) in endplate tissue (L3-L5), benchmarked against the expression of GAPDH, in aged mice treated with PTH or Veh for durations of two weeks, one month, and two months. (n = 5).

**Supplementary Figure 3. Validation of PPR_OCN_^-/-^ mice. (a)** Representative images of PPR positive cells in IVD, OCN^+^ PPR^+^ cells in EP and TB of vertebral body of WT control and PPR_OCN_^-/-^ mice. Scale bar: 100 µm. White arrow: PPR^+^OCN^+^ cells. Yellow arrow: PPR^+^ cells. **(b-d)** Quantitative analysis of PPR positive cells in IVD (b), OCN^+^ PPR^+^ cells in EP (c) and TB (d) of vertebral body in WT control and PPR_OCN_^-/-^ mice. (n = 3, *t*-test). **(e)** Total spontaneous activity distance traveled in two days of PPR_OCN_^-/-^ LSI mice treated with PTH or Veh for two months. (n ≥ 3, *t*-test). ***P*＜0.01, ****P*＜0.005.

**Supplementary Figure 4. Regulation of Nerve Axon Repellant Factors by PTH. (a-b)** mRNA expression levels of *Sema3a* (a) and *Efnb2* (b) in the lumbar endplate tissue of young mice treated with Veh and aged mice treated with Veh or PTH for one month. (n = 3, one-way ANOVA with Tukey’s multiple comparisons test). **(c-f)** mRNA expression levels of *Bglap* (c), *Col1a1* (d)*, Sp7* (e)*, Runx2* (f) genes in MC3T3 cells exposed to either osteoblast differentiation-inducing medium (stimulated medium, S) or unstimulated medium (US) for three days (3d). (n = 3, *t*-test).  **(g-h)** mRNA expression levels of *Sema3a* (g) and *Efnb2* (h) in MC3T3 cells cultured in osteoblast differentiation-inducing medium (stimulated medium). The cells were treated with Veh or PTH at dosages of 10 nM, 100 nM, or 1000 nM for 3 days. (n = 3, one-way ANOVA with Tukey’s multiple comparisons test). **(i)** mRNA expression levels of Slit3 in primary osteoblasts cultured in the stimulated medium with vehicle or PTH treatment (100 nM, three days). (n = 3, *t*-test). **(j-l)** Representative images showing co-immunostaining for OCN (green) and Slit3 (red) in the lumbar spine sections and quantitative analysis of number of Slit3^+^ cells in trabecular bone (TB) and endplate (EP) of WT young LSI mice treated with PTH or Veh for two months. Scale bar: 100 µm. (n ≥ 5, *t*-test). **P*＜0.05, ***P*＜0.01, ****P*＜0.005, *****P*＜0.001.

**Supplementary Figure 5. The binding sites of PTH-stimulated FoxA2 mediated *Slit3* expression. (a)** Representative electrophoresis bands for the FoxA2 binding site in the *Slit3* promoter region from stimulated MC3T3 cells treated with vehicle or PTH (100 nM) for 3 days. **(b)** Diagram illustrating the various locations and sequences of potential FoxA2 or E47 binding sites on the *Slit3* gene promoter with blue and green shading noting PTH-stimulated binding site.

**Supplementary Figure 6. Validation of Slit3_OCN_^-/-^ mice. (a)** Representative images of Slit3 (green) and OCN (cyan) positive cells in the lumbar vertebral body and endplate in WT control and Slit3_OCN_^-/-^ mice. **(b-c)** Quantification of Slit3^+^ cells in trabecular bone (b) and endplate (c) in WT control and Slit3_OCN_^-/-^ mice. Scale bar: 100 µm. (n = 3, *t*-test). **(d-f)** Representative images (d) and quantifications of OCN^+^PPR^+^ cells in trabecular bone (e) and endplate (f) in WT control and Slit3_OCN_^-/-^ mice. Scale bar: 100 µm. (n = 3, *t*-test). **(g-k)** Representative images (g) and quantification of the bone volume per tissue volume (BV/TV) and total porosity percentage of vertebral body (h and i) and endplate (j and k) in WT and Slit3_OCN_^-/-^ mice. Scale bar: 1 mm. (n ≥ 4, *t*-test). **P*＜0.05, ***P*＜0.01, ****P*＜0.005.

**Supplementary Figure 7. The mechanism of PTH induces Slit3 to reduce sensory nerves and relieve pain. (a)** The schematic diagram demonstrates PTH increases transcriptional expression and secretion of Slit3 via FoxA2 in osteoblasts/osteocytes, decreasing sensory nerves in the degenerated vertebral body and endplate, providing pain relief.
