# Supplementary figures and images for: PTH induced osteoblast Slit3 to decrease aberrant sensory innervation in degenerated vertebral endplates to relieve low back pain in mice
Source: Bone Res. 2026 Jan 22;14:12. doi: 10.1038/s41413-025-00488-z (PMC12827982; doi:10.1038/s41413-025-00488-z)

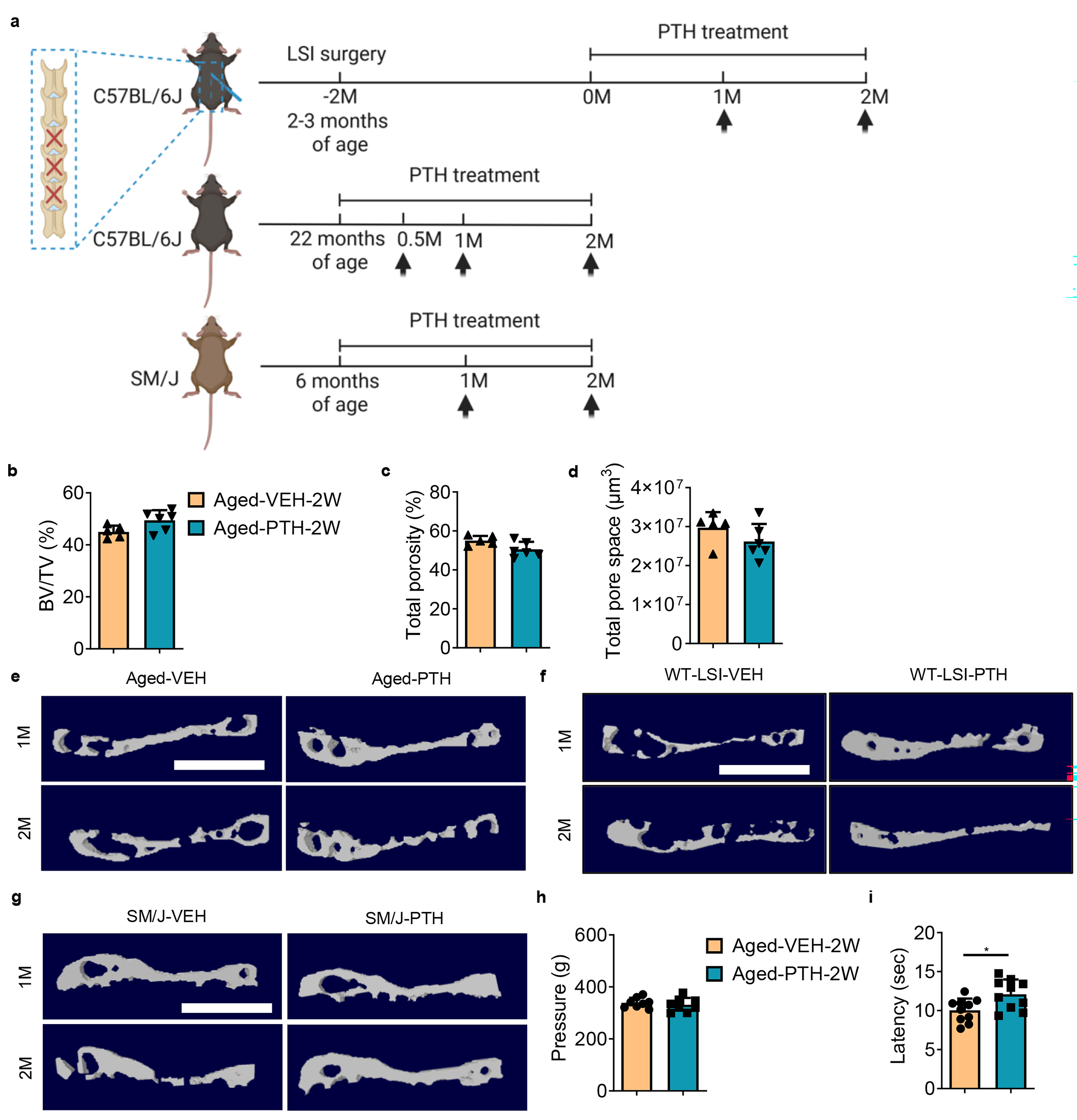

Supplement: Supplementary file 2 — supplementary figure 1 [file 41413_2025_488_MOESM2_ESM.tif]

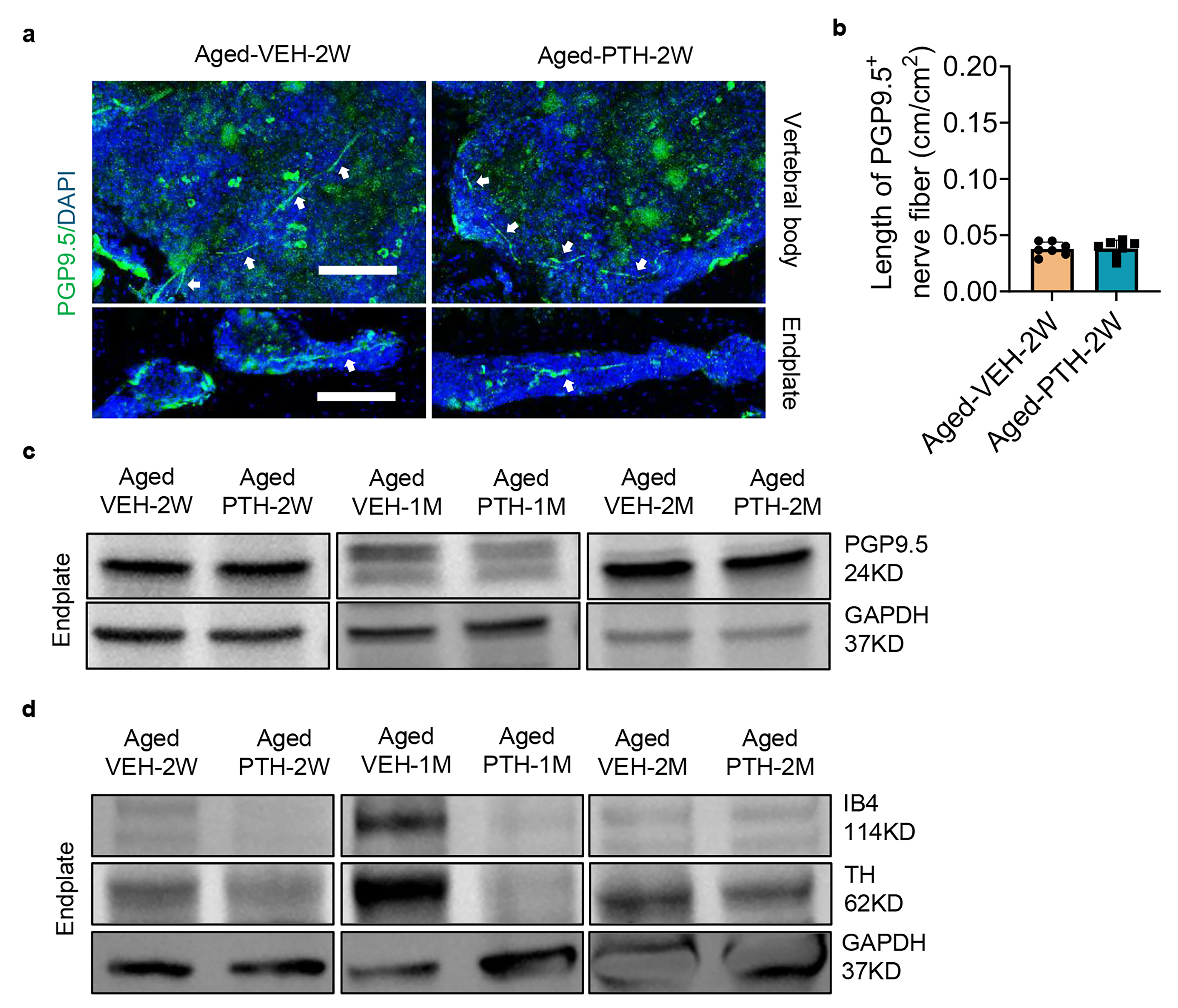

Supplement: Supplementary file 3 — supplementary figure 2 [file 41413_2025_488_MOESM3_ESM.tif]

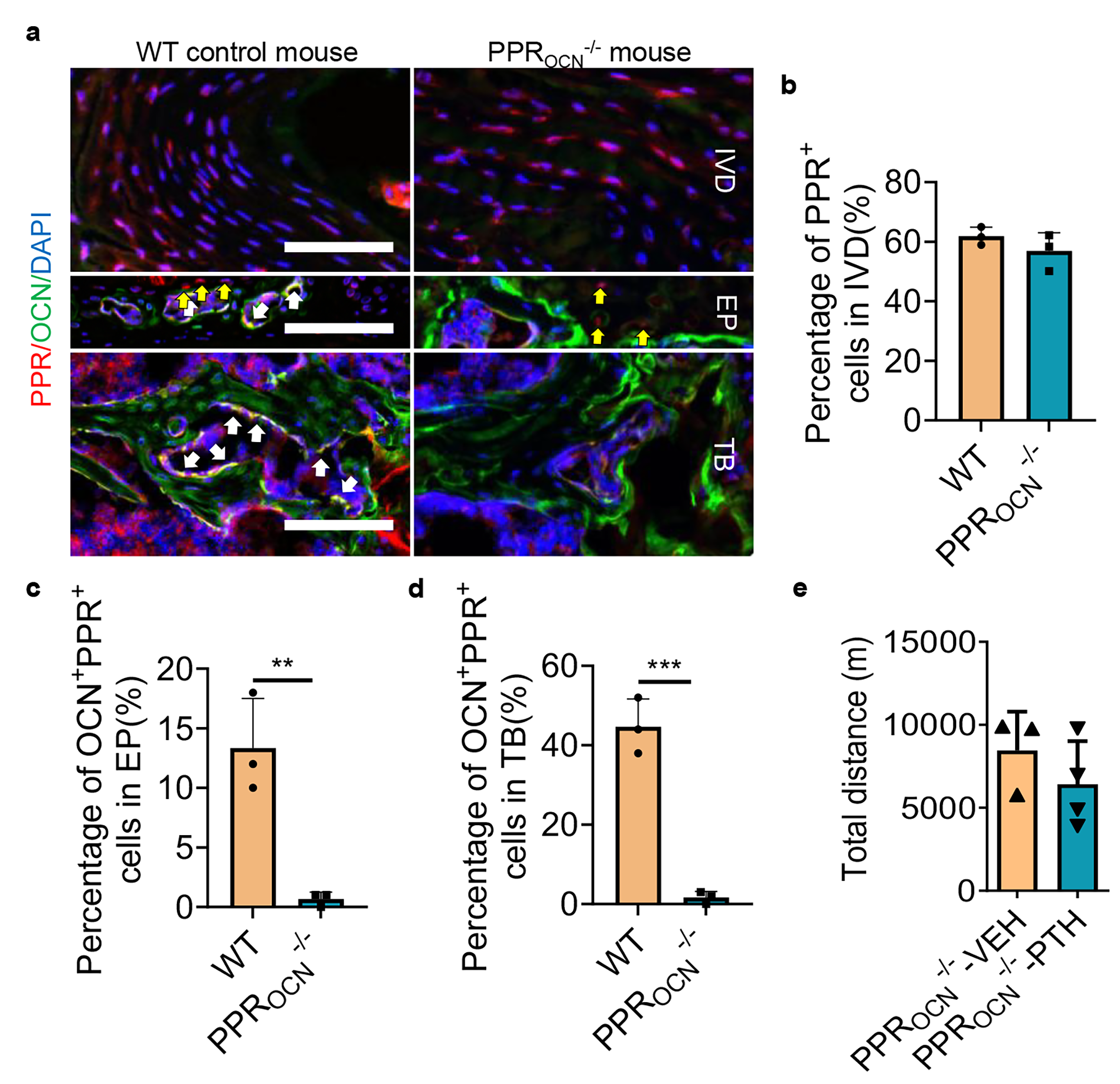

Supplement: Supplementary file 4 — supplementary figure 3 [file 41413_2025_488_MOESM4_ESM.tif]

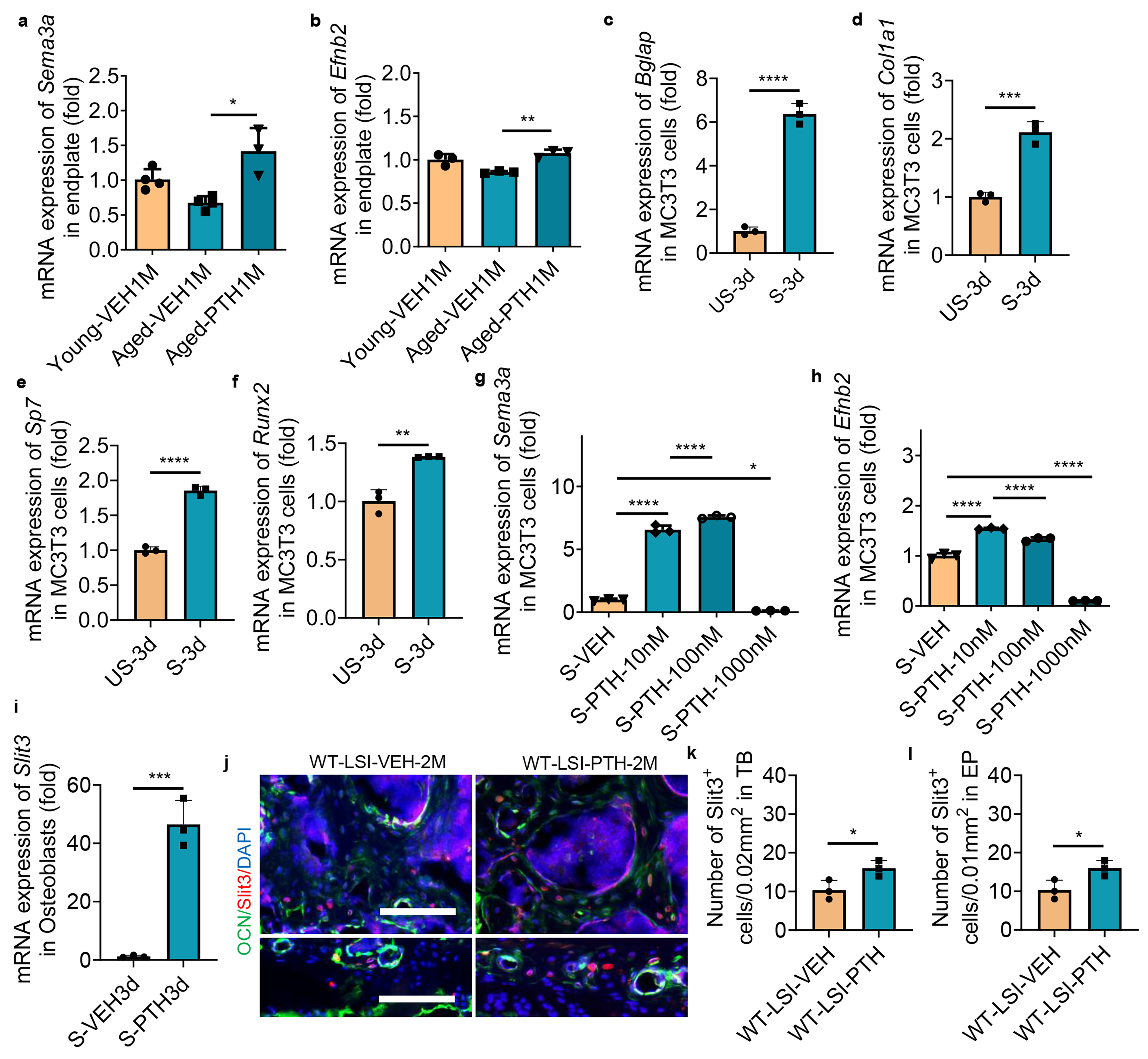

Supplement: Supplementary file 5 — supplementary figure 4 [file 41413_2025_488_MOESM5_ESM.tif]

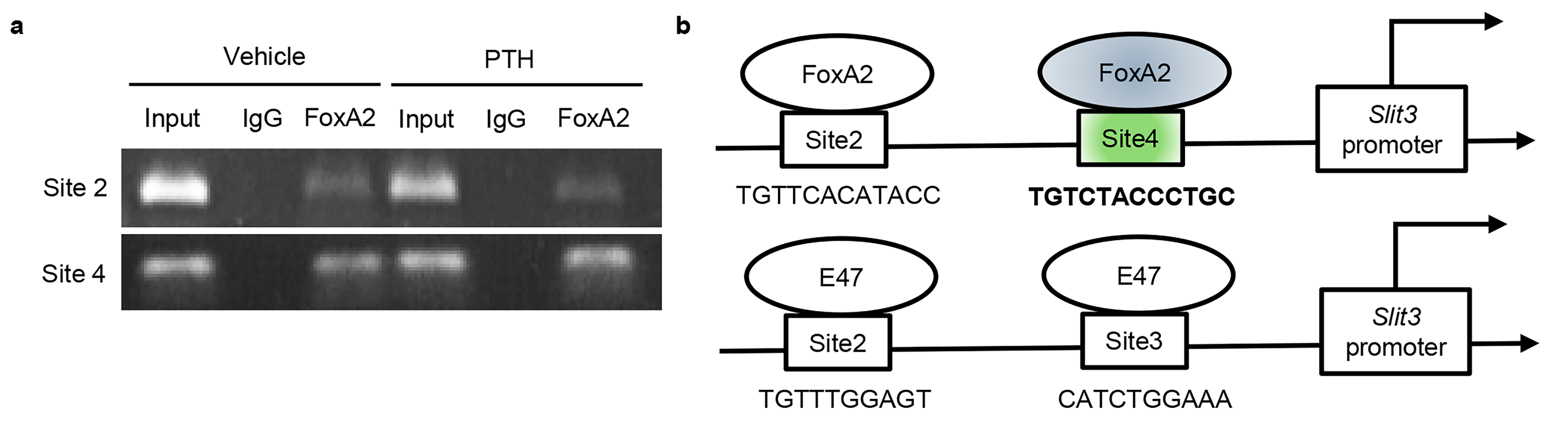

Supplement: Supplementary file 6 — supplementary figure 5 [file 41413_2025_488_MOESM6_ESM.tif]

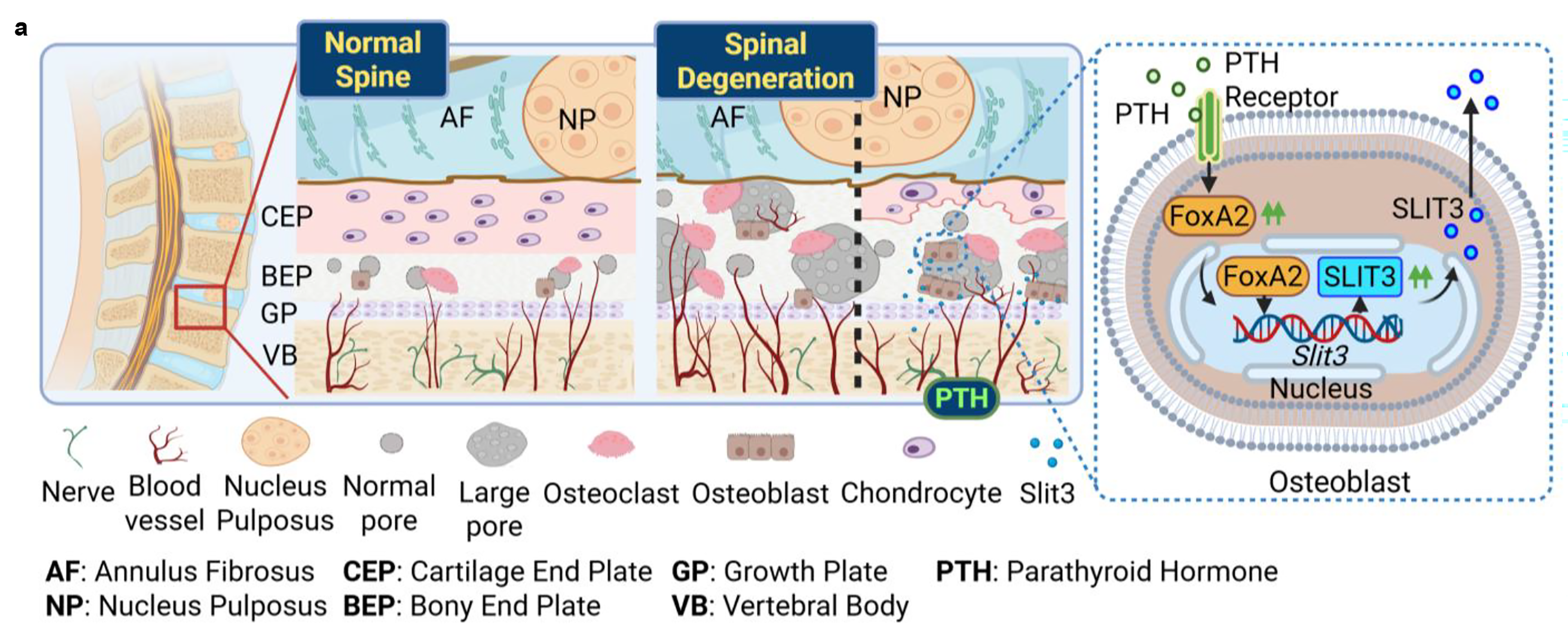

Supplement: Supplementary file 8 — supplementary figure 7 [file 41413_2025_488_MOESM8_ESM.tif]

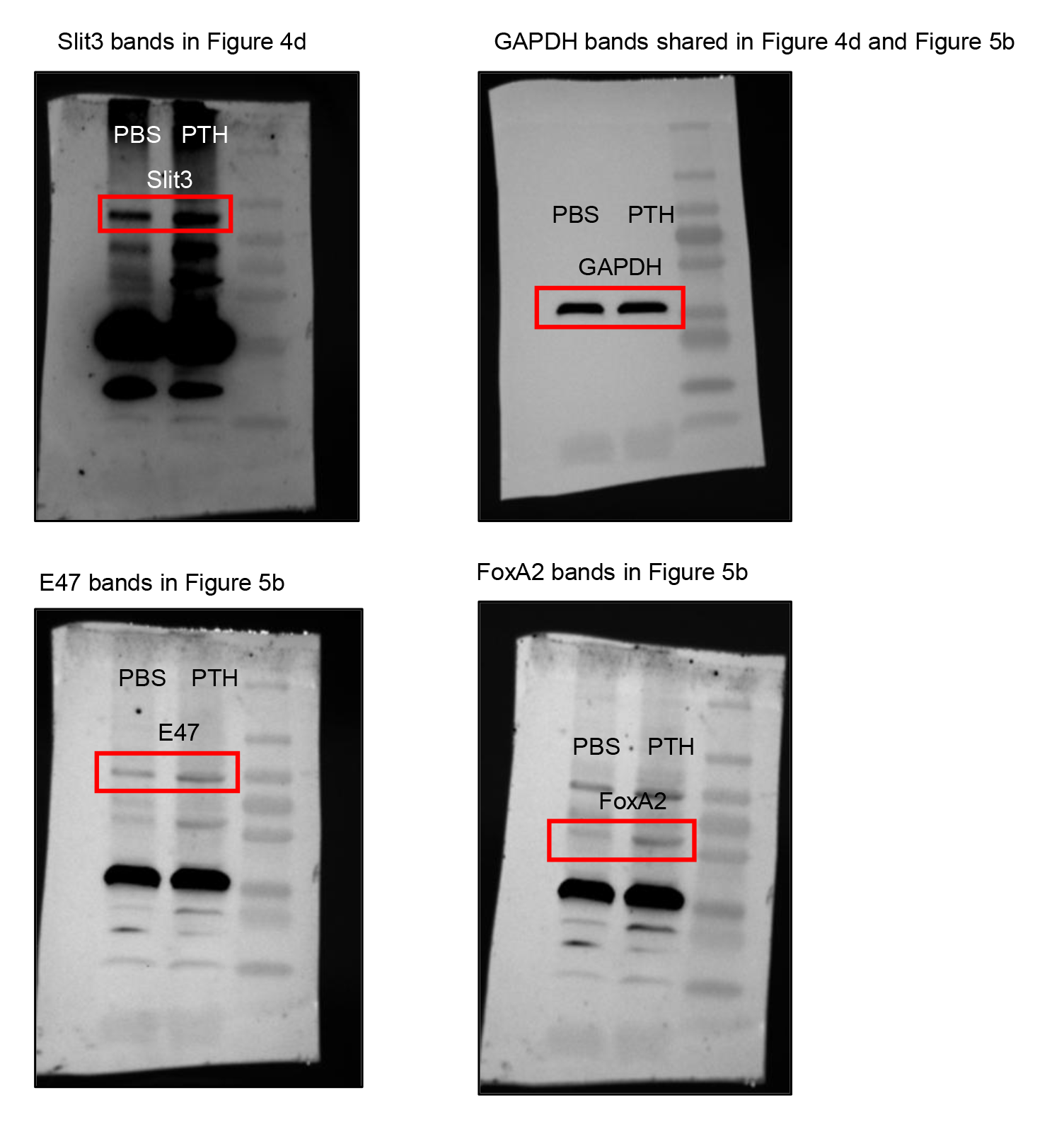

Supplement: Supplementary file 9 — original bands of WB in Figure 4d and Figure 5b [file 41413_2025_488_MOESM9_ESM.tif]
